# Scutellarin attenuates high glucose-induced edema and inflammation in retinal Müller cells via AQP4/Kir4.1 restoration and TLR4/MyD88/NF-κB inhibition

**DOI:** 10.3389/fphar.2026.1923023

**Published:** 2026-09-18

**Authors:** Yunhua Tang, Shan Ding, Wenyan Peng, Meichu Wu, Yongqi Mao, Wenxin Yang, Xiaoyang Li, Fuwen Zhang

**Affiliations:** 1 Eye School of Chengdu University of TCM, Chengdu, Sichuan, China; 2 Ziyang Hospital of Traditional Chinese Medicine, Ziyang, Sichuan, China; 3 Key Laboratory of Sichuan Province Ophthalmopathy Prevention and Cure and Visual Function Protection with TCM Laboratory, Chengdu, Sichuan, China; 4 Retinal Image Technology and Chronic Vascular Disease Prevention and Control and Collaborative Innovation Center, Chengdu, Sichuan, China; 5 Department of Ophthalmology,Hospital of Chengdu University of TCM, Chengdu, Sichuan, China

**Keywords:** AQP4/Kir4.1, diabetic macular edema, diabetic retinopathy, retinal Müller cells, Scutellarin, TLR4/MyD88/NF-κB/VEGFA

## Abstract

**Background:**

Diabetic retinopathy (DR) is a metabolic eye disease closely linked to inflammation and oxidative stress, potentially causing vision decline or blindness. Scutellarin (SCU), a major flavonoid extracted from Erigeron breviscapus, possesses anti-inflammatory and antioxidant properties; however, its protective mechanisms against retinal Müller cell (RMC) injury in DR remain unclear.

**Objective:**

To investigate the protective effects and underlying mechanisms of SCU against high-glucose(HG)-induced edema and inflammation in retinal Müller cells (RMCs).

**Materials and Methods:**

Primary RMCs were isolated from newborn 3–5-day-old Sprague-Dawley (SD) rats and exposed 100 mmol/L HG to establish an *in vitro* injury model. Groups included control, model (HG), low-dose (HG+25 μmol/L SCU), and high-dose (HG+50 μmol/L SCU). Cell viability, migration capacity, Na^+^-K^+^-ATPase activity, and apoptosis were assessed using CCK-8 assays, scratch wound healing assays, enzymatic activity assays, and flow cytometry, respectively. The expression and distribution of AQP4, Kir4.1, and molecules associated with the TLR4/MyD88/NF-κB/VEGFA pathway were evaluated via immunofluorescence, Western blot, and qRT-PCR.

**Results:**

HG exposure significantly suppressed RMC viability, induced apoptosis, disrupted the functional coupling of AQP4/Kir4.1, and activated the TLR4/MyD88/NF-κB/VEGFA signaling pathway. SCU treatment dose-dependently reversed these pathological changes, restored Na^+^-K^+^-ATPase activity and the expression balance of AQP4/Kir4.1, and inhibited the excessive activation of the inflammatory pathway.

**Conclusion:**

The protective effects of SCU are significantly associated with the restoration of AQP4/Kir4.1 homeostasis and the suppression of the TLR4/MyD88/NF-κB/VEGFA pathway, suggesting its potential as a multi-target candidate drug for delaying the progression of DR.

## 1 Introduction

Diabetic retinopathy (DR) is a metabolic disease that is a leading cause of blindness among people of working age (Antonetti et al., 2012), and diabetic macular edema is the primary cause of vision loss associated with DR (Ng et al., 2025). Epidemiological data indicate that there were approximately 103.1 million people with DR worldwide in 2020, a number projected to reach 160 million by 2045; during the same period, the number of people with diabetic macular edema is expected to rise to 29 million (Teo et al., 2021). The core pathophysiology of DR involves metabolic disorders, oxidative stress, and inflammation, which lead to dysfunction of the retinal neurovascular unit (NVU) (Liu et al., 2022). Damage to the NVU leads to capillary occlusion and increased ischemic burden, which disrupts the blood-retinal barrier (BRB), further resulting in vascular leakage, neovascularization, and fibrovascular proliferation (Nian et al., 2021). Retinal Müller cells (RMCs), as the core of the NVU, are rich in ion channels, ligands, receptors, transmembrane transporters, and enzymes that regulate the balance of ions and water across the cell membrane and maintain the retinal homeostasis (Lai et al., 2023).

Currently, conventional treatments for DR primarily target retinal microvascular degeneration in the middle-to-late stages of the disease (Li and Zhou, 2025). These include laser photocoagulation, anti-vascular endothelial growth factor (VEGF) drugs, glucocorticoids, and vitrectomy (Tomita et al., 2021). These approaches struggle to reverse established microvascular pathological damage and are subject to limitations such as the need for repeated treatments, surgical complications, suboptimal efficacy in some patients, and high treatment costs (Seo et al., 2025), which severely compromise patients’ long-term adherence and overall prognosis. It is worth noting that the potential reversibility of early-stage DR lesions has not received sufficient attention. NVU damage may be the core initiating factor in the onset of asymptomatic early-stage DR (Das, 2016), and RMC occurs in conjunction with retinal neuronal apoptosis; an imbalance in RMC functional homeostasis has been identified as a key pathological feature of early-stage DR (Yang et al., 2022). Therefore, there is an urgent need to develop novel intervention strategies that are long-acting, convenient, and enable personalized, precision treatment to prevent and treat early-stage DR by protecting the NVU, thereby improving patients’ long-term prognosis.

Our research team previously formulated the traditional Chinese medicine compound “Qidengmingmu Capsule (QD),” which contains Erigeron breviscapus, Astragalus membranaceus (Fisch.) Bunge, and Pueraria montana (Lour.) Merr, which shows potential therapeutic value for treating DR. Our previous animal studies have demonstrated that QD can inhibit hyperoxia-induced retinal neovascularization in mice, promote the maturation and remodeling of new blood vessels, and exert a retinal protective effect via the Ang/Tie2 signaling pathway (Liu et al., 2026). Erigeron breviscapus has garnered significant attention for its therapeutic value in ophthalmic diseases. In glaucoma models, it exerts neuroprotective effects by inhibiting retinal ganglion cell apoptosis and improving optic disc blood circulation (Wu et al., 2021; Cheng et al., 2024; Yang et al., 2025). Other natural metabolites have also demonstrated considerable potential. For instance, Brazilin-enriched Caesalpinia sappan heartwood has emerged as a promising natural adjunct therapy for cardioprotection by modulating oxidative stress and mitigating tissue damage *in vivo* (Singaravel et al., 2025). Furthermore, Sanggenon L, a flavonoid isolated from Morus alba root bark, has been reported to protect retinal ganglion cells against retinal ischemia/reperfusion injury by alleviating oxidative stress, inflammation, and apoptosis (Xu et al., 2026). Scutellarin (SCU), the major flavonoid derived from Erigeron breviscapus (Wang et al., 2023), is recognized as a key bioactive metabolite of QD (Liu et al., 2021). SCU exhibits a broad spectrum of pharmacological activities, including antioxidant, anti-inflammatory, and neuroprotective effects, as well as the inhibition of pathological angiogenesis and tumorigenesis (Wang and Ma, 2018; Nie et al., 2024; Xie et al., 2024). In glaucoma models, SCU has been shown to protect retinal ganglion cells and suppress their apoptosis (Su et al., 2025). In the context of DR, SCU has been demonstrated to inhibit cellular apoptosis, alleviate inflammatory damage, preserve the BRB, and suppress pathological neovascularization (Zhu et al., 2018; Mei et al., 2019; Wang et al., 2021). Further *in vivo* studies in diabetic rats by Yang et al. (2024) revealed that SCU mitigates retinal inflammation and neurodegeneration by inhibiting the NLRP3 inflammasome. Additionally, Long et al. (2019) reported that the anti-angiogenic effects of SCU are mediated through the suppression of the expression and interactions among VEGF, p-ERK, p-FAK, and p-Src. Our previous research has also established that SCU regulates the VEGF/Ang/Tie2 signaling axis in rat retinal microvascular endothelial cells, thereby inhibiting pathological angiogenesis and improving vascular stability under hypoxic conditions (Ding et al., 2025). However, given that RMCs dysfunction is recognized as an early event in DR pathogenesis (Yang et al., 2022), it remains unclear whether SCU can directly target RMCs to maintain retinal homeostasis and alleviate inflammatory injury. The precise molecular mechanisms underlying these potential effects warrant further investigation.

Building upon the current research landscape, this study established an *in vitro* high glucose (HG)-induced RMC injury model. We aimed to investigate whether SCU inhibits the activation of the TLR4/MyD88/NF-κB/VEGFA signaling pathway and to evaluate its effects on AQP4/Kir4.1 water-ion coupling and Na^+^-K^+^-ATPase activity in RMCs. By elucidating the cellular and molecular targets of SCU, this study provides experimental evidence supporting its potential as a multi-target therapeutic candidate for delaying DR progression, specifically through the maintenance of retinal water and ion homeostasis, the mitigation of inflammatory injury, and the exertion of cytoprotective functions.

## 2 Materials and methods

### 2.1 Animals

The newborn 3–5-day-old SPF Sprague-Dawley rats (both sexes, weighing approximately 7–10 g) used in the experiment were provided by the Animal Laboratory of Chengdu University of TCM. In alignment with the principles of Replacement, Reduction, and Refinement (the 3Rs), all animal protocols followed established international welfare standards to ensure minimal distress and the lowest feasible animal usage.

### 2.2 Reagents and chemicals

The reagents and chemicals used in this study are as follows: SCU (Chengdu Aifa Biotech, China, CAS: 27740-01-8), D-Glucose (anhydrous, BioFroxx, China, 1179GR500), Mannitol injection (Sichuan Kelun Pharmaceutical, China), DMEM (HG, Gibco, United States, Cat. No. C11995500BT), Penicillin-Streptomycin solution (Pen-Strep, 100×, Hyclone, United States, Cat. No. SV30010), FBS (Senbeijia, China, Cat. No. BC-SE-FBS01), D-Hanks buffer (Solarbio, China, Cat. No. H1045), 0.25% Trypsin-EDTA (Hyclone, United States, Cat. No. SH30042.01), PBS (pH 7.2–7.4, Solarbio, China, Cat. No. P1020), CCK-8 (Abmole, United States, Cat. No. C0038), 5%Triton X-100 permeabilization solution (BioFroxx, Germany, Cat. No. 1139ML500), Bovine serum albumin (Servicebio, China, Catalog No. GC305010), FITC-Tyramide (Servicebio, China, Cat. No. G1222-50UL), Goat anti-Rabbit IgG (MultiSciences, China, Cat. No. GAR0072), DAPI staining solution (Servicebio, China, Cat. No. G1012), Fluoromount-G (SouthernBiotech, United States, Cat. No. 0100-01), RIPA Lysis Buffer (Beyotime, China, Catalog No. P0013B), Trypsin digestion solution (0.25%, without EDTA and phenol red) (Solarbio, China, Catalog No. T1350), SDS-PAGE protein loading buffer (5×, reducing) (Biosharp, China, Catalog No. BL502B), TBST buffer (1×, ready-to-use, containing Tween 20) (Biosharp, China, Catalog No. BL609A), Skimmed milk powder (blotting grade) (Servicebio, China, Catalog No. GC310001-100 g), Enhanced chemiluminescence substrate (Beyotime, China, Catalog No. P0018S).

### 2.3 Cell isolation, culture, and identification

#### 2.3.1 RMCs isolation and culture

Cells were grown in complete medium containing 79% modified DMEM, 20% FBS, and 1% Pen-Strep (100×), and maintained in a humidified 37 °C incubator equilibrated with 5% CO_2_.

All procedures were performed under aseptic conditions. Rats were euthanized by cervical dislocation, and their eyes were removed after soaking the entire body in 75% ethanol for 5 min. The eyes were then immersed in pre-chilled D-Hank’s solution, followed by isolation of the retinal tissue. The retinal tissue was incubated with 0.25% trypsin-EDTA in an incubator for 5 min, then digestion was terminated with complete medium. After two centrifugations (1,000 rpm, 5 min), the cell pellet was collected. Following centrifugation, cells were reconstituted in complete medium and seeded at 2.5 × 10^4^ cells/cm^2^ into tissue culture-treated dishes. RMCs were cultured in a 37 °C/5% CO_2_ incubator, with biweekly medium exchanges. Once cell confluence reached 90% or higher, the cells were passaged; the second-generation (P2) cells were used for subsequent experiments. For details on the culture system used in this experiment, refer to the published literature (Tang et al., 2025).

#### 2.3.2 Hematoxylin and eosin (H&E) staining

Using the H&E staining kit (Solarbio, China, G1120), samples were fixed in 4% paraformaldehyde (15 min), rinsed in PBS, and subjected to hematoxylin (15 min) and eosin (2 min) staining. Dehydration was performed via an ascending ethanol gradient (75% → 100%), after which slides were mounted in neutral resin and examined.

#### 2.3.3 Immunofluorescence staining

Immunofluorescence staining was performed to detect intracellular target protein expression. Cell smears from RMC-infected cultures were washed three times with PBS (3 min each), fixed in 4% paraformaldehyde for 15 min at room temperature, briefly rinsed with PBS, and permeabilized for 10 min at room temperature with 0.5% Triton X-100 in PBS. Following another PBS wash, samples were blocked with 3% BSA in PBS for 20 min at room temperature, then incubated overnight at 4 °C with primary antibodies diluted as indicated (Supplementary Table S1). The next day, after thorough PBS washing, slides were incubated with FITC-conjugated goat anti-rabbit IgG (1:200) for 50 min at room temperature in the dark, counterstained with DAPI for 10 min (room temperature, dark), washed again, and mounted with anti-fade mounting medium (Fluoromount-G). All fluorescence-related steps were conducted under light-protected conditions, and images were acquired using a fluorescence microscope.

### 2.4 Cell viability assay

The CCK-8 assay was employed to evaluate RMC viability under varying glucose concentrations and SCU treatment. Glucose solutions (0–1,000 mmol/L) were prepared in sterile PBS, with isotonic mannitol included as an osmotic control. P2 RMCs were dissociated with 0.25% trypsin-EDTA, centrifuged, resuspended in complete medium, and seeded into 96-well plates at 1 × 10^4^ cells/well, followed by 24 h of standard culture. Cells were then exposed to graded glucose (0, 1, 3, 10, 30, 100, 300, 1,000 mmol/L) or SCU (0, 25, 50, 100, 200, 400, 800 μmol/L) for 24 h or 48 h, respectively, with matched mannitol controls. Post-treatment, 10 μL of CCK-8 reagent was added per well, and plates were incubated in the dark. Absorbance at 450 nm was measured at 0.5, 1, 1.5, and 2 h using a microplate reader (Multiskan FC; Thermo Fisher Scientific, United States), with values normalized to cell-free blanks to calculate relative viability. Based on pilot data, a DR *in vitro* model was established using 100 mmol/L glucose for 24 h, and SCU doses of 25 and 50 μmol/L were selected for subsequent studies. The final experimental groups were: control (complete medium), model (100 mmol/L glucose, 24 h), low-dose (100 mmol/L glucose +25 μmol/L SCU, 24 h), and high-dose (100 mmol/L glucose +50 μmol/L SCU, 24 h).

### 2.5 Measurement of Na^+^-K^+^-ATPase

Na^+^-K^+^-ATPase activity was measured using the Solarbio assay kit (Cat. No. BC0065) per manufacturer’s instructions. Cells from each group were washed with PBS, lysed on ice, and centrifuged at 4 °C; supernatants were collected and protein concentrations determined by BCA assay. Equal amounts of protein were mixed with reaction buffer and ATP to initiate the enzymatic reaction, which was incubated at 37 °C and then terminated. The released inorganic phosphate was quantified spectrophotometrically. A standard curve was generated using phosphate standards, and absorbance was read at 562 nm on a microplate reader. Duplicate wells were averaged, and enzyme activity in test samples was calculated using the standard curve regression equation.

### 2.6 Flow cytometry apoptosis detection experiment

Apoptosis in RMCs from each group was assessed using the Annexin V-FITC/PI kit (MultiSciences, Cat. No. AP-101-100-kit). Culture supernatants were collected, and adherent cells were detached with EDTA-free trypsin. Cells and supernatants were pooled, washed twice with ice-cold PBS, and centrifuged (1,500 rpm, 5 min). The pellet was resuspended in 1× Binding Buffer, followed by sequential addition of Annexin V-FITC and PI, and incubated at room temperature in the dark for 5 min. Unstained, Annexin V-FITC-only, and PI-only controls were prepared in parallel for flow cytometer voltage setting and fluorescence compensation. Samples were analyzed immediately by flow cytometry to determine the percentage of apoptotic cells per group.

### 2.7 Western blot

Western blotting was performed to assess protein expression across groups. Cells were harvested, washed three times with ice-cold PBS, lysed on ice for 60 min in RIPA buffer supplemented with protease and phosphatase inhibitors, then centrifuged (12,000 rpm, 10 min); supernatants were collected. Protein concentrations were quantified by BCA assay. Equal amounts of protein were mixed with 5× SDS loading buffer and denatured by boiling. Samples (20 μg) were resolved by SDS-PAGE and transferred onto PVDF membranes via wet transfer. Membranes were blocked with 5% non-fat milk in TBST for 1 h at room temperature, washed three times with TBST, and incubated overnight at 4 °C with primary antibodies (details in Supplementary Table S2). After washing, membranes were probed with HRP-conjugated goat anti-rabbit IgG (1:1,000) for 1 h at room temperature, washed again, and developed using enhanced chemiluminescence (ECL). Band intensities were quantified using ImageJ software.

### 2.8 mRNA isolation and quantitative polymerase chain reaction

Total RNA was isolated from RMCs in each group using the FastPure Cell Total RNA Isolation Kit V2 (Vazyme, Cat. No. RC112-01). Equal amounts of RNA were reverse-transcribed into cDNA in 20 μL reactions using the manufacturer’s protocol (Vazyme, Cat. No. R333-01), which includes on-column genomic DNA removal and reverse transcription (50 °C for 15 min, 85 °C for 5 s). The resulting cDNA was dispensed into PCR 8-tube strips, briefly centrifuged to eliminate air bubbles, and subjected to real-time quantitative PCR. Amplification conditions: initial denaturation at 95 °C for 30 s, followed by 45 cycles of 95 °C for 10 s and 60 °C for 30 s, with a subsequent melt curve analysis. GAPDH served as the internal reference, and gene expression in the control group was normalized to 1. Primer sequences are provided in Supplementary Table S3.

### 2.9 Statistical analysis

Statistical analyses were performed using SPSS 26.0. Quantitative data are presented as mean ± SD. The sample size (n) refers to the number of independent biological replicates. Normality was assessed using the Shapiro-Wilk test. For normally distributed data with homogeneous variances (Levene’s test, P > 0.05), one-way ANOVA followed by Tukey’s *post hoc* test was used. When variances were heterogeneous (Levene’s test, P < 0.05), Welch’s ANOVA followed by Games-Howell *post hoc* test was applied. Statistical significance was defined as P < 0.05. All experiments were independently repeated at least three times. Data visualization was generated with GraphPad Prism 10.1.2.

## 3 Results

### 3.1 Identification of RMCs

Under an inverted light microscope, RMCs appear spindle-shaped overall, with a well-defined cell body, abundant cytoplasm, and a round or oval nucleus, from which characteristic cellular projections extend outward (Figure 1A). H&E staining reveals that the cytoplasm appears pink and the nucleus appears blue-violet, with intact cell morphology and clear structure (Figure 1C). Immunofluorescence staining results indicate that DAPI-labeled nuclei exhibit blue fluorescence; the cytoplasmic-specific marker Glutamine Synthetase (GS) exhibits red fluorescence, while CRALBP and vimentin exhibit green fluorescence; AQP4 and Kir4.1 are primarily localized to the cell membrane and exhibit red and green fluorescence, respectively. RMC cell bodies are mostly round, oval, or irregular in shape, with clear boundaries; the fluorescence signal in the cell process regions is relatively weak (Figure 1D). Flow cytometry was used to assess cell purity using GS- and CRALBP-specific antibodies; the results showed that the purity of the RMCs isolated and cultured in this experiment reached over 95% (Figure 1B).

**FIGURE 1:**
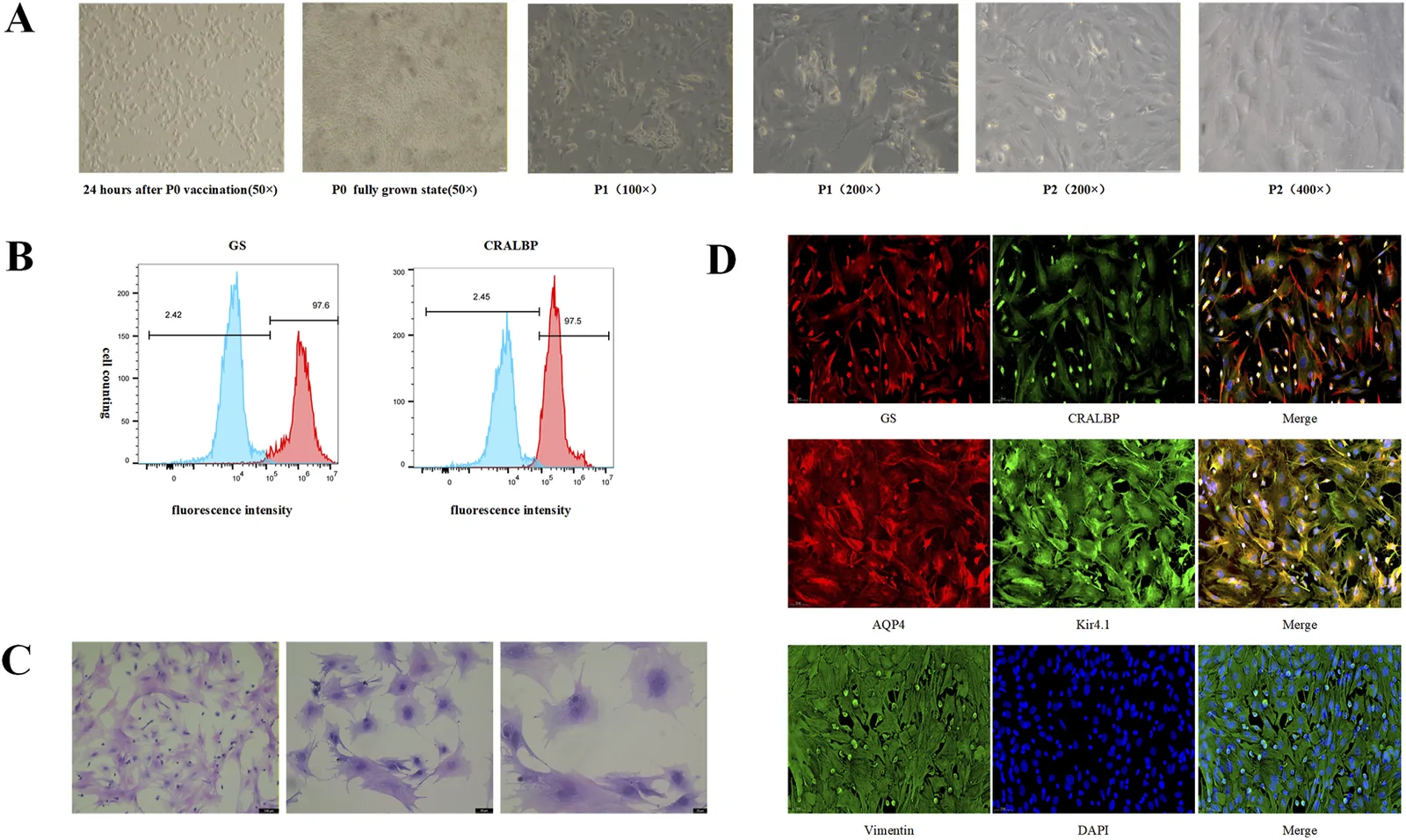
Results of RMC characterization. **(A)** Morphology of RMCs at different culture stages under an inverted light microscope at various magnifications. From left to right: cells 24 h after seeding (Passage 0, P0); P0 cells at confluence; P1 cells; and P2 cells. The cells are generally spindle-shaped with well-defined cell bodies and visible protrusions, exhibiting typical morphological characteristics of RMCs. Scale bar: 100 μm. **(B)** Flow cytometry analysis of GS and CRALBP expression showed a purity of >95%. **(C)** H&E staining shows intact cell morphology with pink cytoplasm and blue-violet nuclei; scale bars are 100 μm, 50 μm, and 20 μm, respectively. **(D)** Immunofluorescence staining reveals positive expression of GS, AQP4, CRALBP, Kir4.1, and vimentin; DAPI labels the nuclei. Scale bar: 50 μm. Part of this figure was adapted from Tang et al. (2025).

### 3.2 Conditions for high-glucose modeling and SCU intervention concentrations

Figure 2 shows the cell viability results after 24-h and 48-h treatments with glucose and SCU at different concentrations, with an isotonic mannitol solution serving as the osmotic control. Based on preliminary results, subsequent experiments employed 100 mmol/L glucose for 24 h to induce the RMC model, with SCU administered at 25 μmol/L (low dose) and 50 μmol/L (high dose) for 24 h.

**FIGURE 2:**
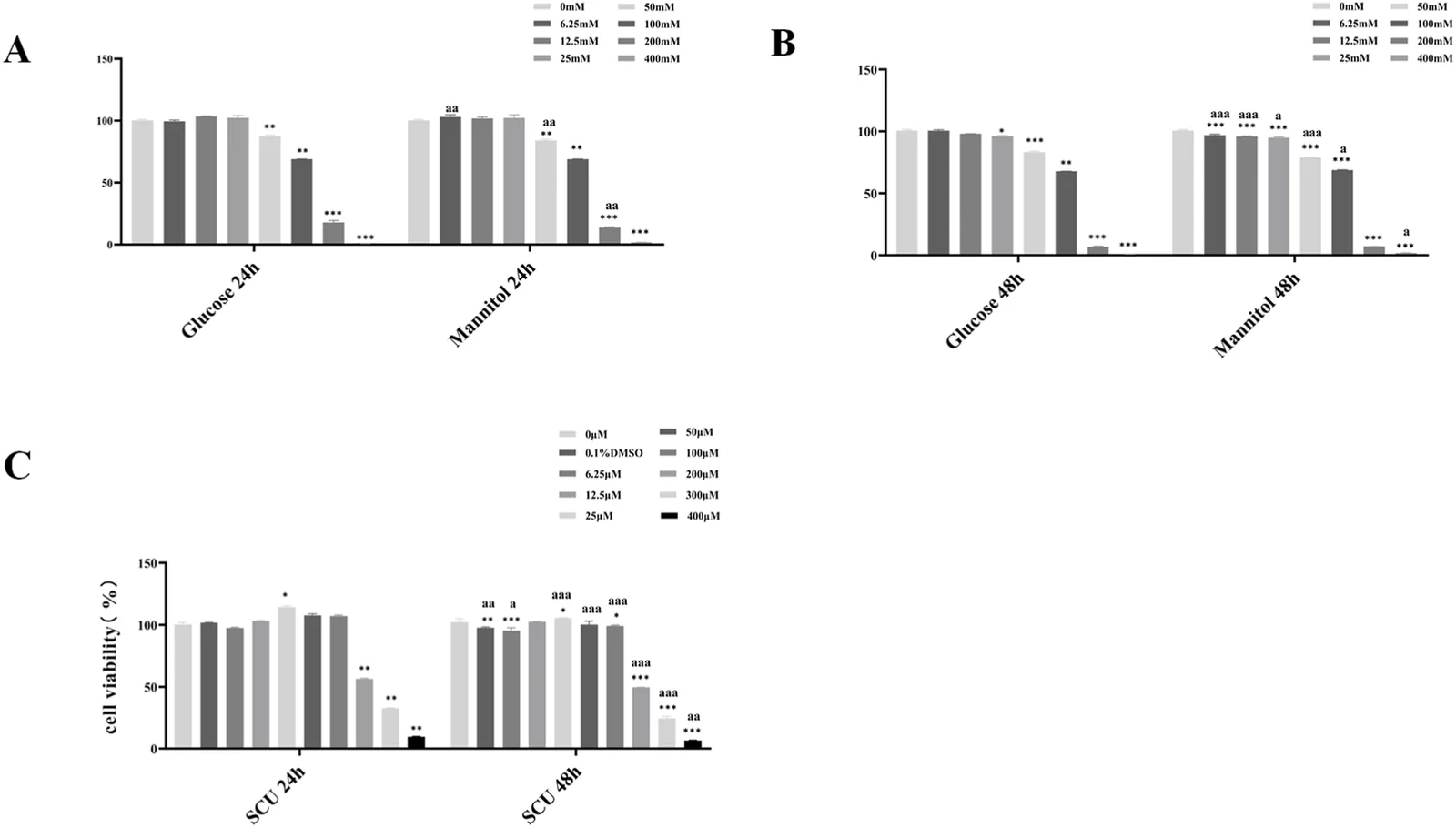
Cell viability 24 and 48 h after glucose and SCU treatment, respectively. **(A,B)** RMC viability after 24 h and 48 h exposure to varying glucose concentrations, with isotonic mannitol as an osmotic control. Note: *P < 0.05, **P < 0.01, ***P < 0.001 vs. 0 mmol/L group; ^a^ *P* < 0.05, ^aa^ *P* < 0.01, ^aaa^ *P* < 0.001 vs. mannitol control at the same concentration. **(C)** RMCs viability after 24 h and 48 h treatment with increasing SCU concentrations. Note: *P < 0.05, **P < 0.01, ***P < 0.001 vs. 0 μmol/L group; ^a^ *P* < 0.05, ^aa^ *P* < 0.01, ^aaa^ *P* < 0.001 for 24 h vs. 48 h at the same SCU concentration.

### 3.3 SCU inhibits cell migration in RMCs induced by HG

Scratch assay results (Figure 3) showed that cell migration increased progressively over time in all groups, with rates at 24 h significantly higher than those at 6 h and 12 h within each group (*P* < 0.05). At 24 h, the model group exhibited significantly greater migration than the control (*P* < 0.05), whereas neither the low- nor high-dose SCU groups differed significantly from the control at any time point (6, 12, or 24 h; *P* > 0.05). However, both SCU-treated groups showed significantly reduced migration compared to the model group at 24 h (*P* < 0.05). These findings indicate that SCU effectively inhibits HG-induced hypermotility, suggesting that it may interfere with the behavioral alterations associated with glial activation.

**FIGURE 3:**
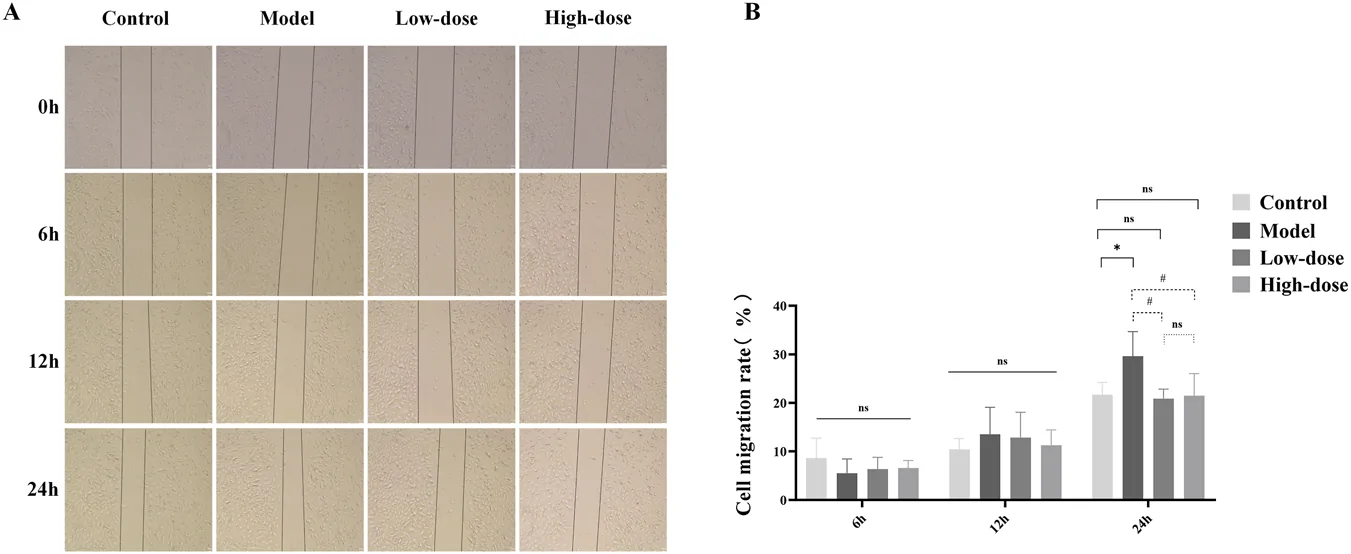
Effect of SCU on HG–induced RMC migration. **(A)** Representative phase-contrast images from the scratch assay showing RMC migration in control, model, low-dose, and high-dose groups at 0, 6, 12, and 24 h. Scale bar: 50 μm. **(B)** Quantification of cell migration rates across groups and time points. Note: *P < 0.05 vs. control group; ^#^ *P* < 0.05 vs. model group.

### 3.4 SCU inhibits high-glucose-induced inactivation of NA^+^-K^+^-ATPase and apoptosis in RMCs

Observation under an inverted optical microscope revealed that RMCs in the control group exhibited normal morphology; in contrast, RMCs in the model group showed marked swelling, increased volume, blurred cell margins, and reduced cell density following treatment with 100 mmol/L glucose. Compared with the model group, cell morphology in both the low-dose group (25 μmol/L SCU) and the high-dose group (50 μmol/L SCU) showed significant improvement, suggesting that SCU can effectively counteract hyperglycemia-induced cytotoxic damage and maintain normal RMC morphology (Figure 4A).

**FIGURE 4:**
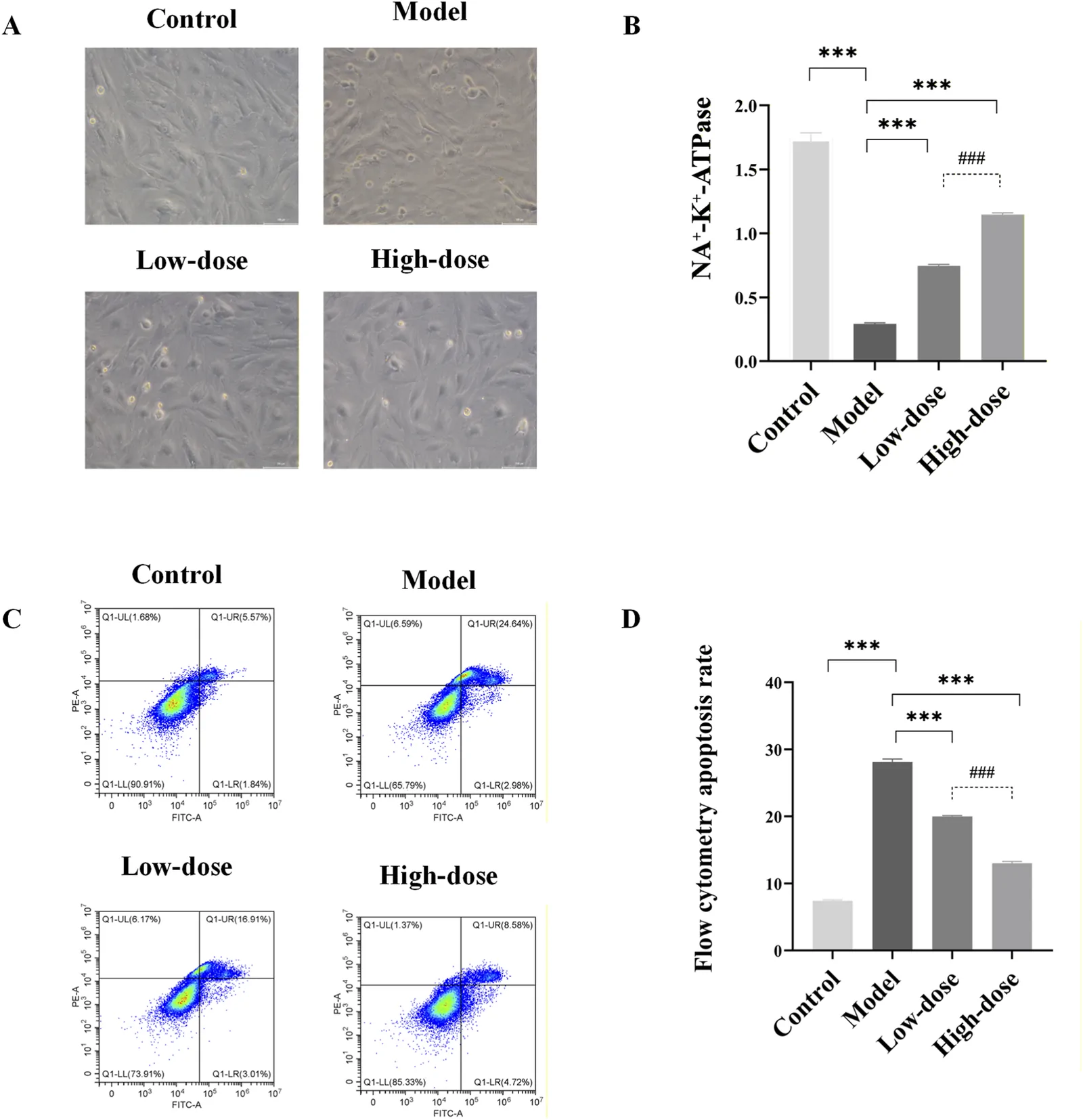
Effects of SCU on RMC Na^+^-K^+^-ATPase inactivation and apoptosis. **(A)** Representative images of RMC cell morphology in each group under an inverted light microscope; scale bar: 100 μm. **(B)** Na^+^-K^+^-ATPase activity was markedly suppressed in the model group and restored by SCU in a dose-dependent manner. **(C)** Annexin V-FITC/PI flow cytometry dot plots (x-axis: FITC-A; y-axis: PE-A) showing reversal of HG–induced apoptosis by SCU. **(D)** Quantification of apoptosis rates. Note: ***P < 0.001 vs. control; ^###^ *P* < 0.001 vs. low-dose group.

Na^+^-K^+^-ATPase activity was markedly reduced in the model group versus control (*P* < 0.001); SCU treatment restored activity in a dose-dependent manner, with both doses significantly exceeding the model group (*P* < 0.001) (Figure 4B).

Flow cytometry revealed a significant increase in RMCs apoptosis under HG (*P* < 0.001 vs. control), which was attenuated by SCU. The high-dose group exhibited lower apoptosis than the low-dose group (*P* < 0.001), confirming a dose-dependent anti-apoptotic effect (Figures 4C,D).

### 3.5 SCU maintains water and ion homeostasis in RMC under high-glucose conditions

Immunofluorescence staining (Figure 5) revealed that, compared to the model group, AQP4 protein expression was significantly downregulated in the control, low-dose, and high-dose groups (all *P* < 0.001), whereas Kir4.1 fluorescence intensity was markedly increased (control and high-dose: *P* < 0.001; low-dose: *P* < 0.05). Within SCU-treated groups, AQP4 expression was further reduced in the high-dose versus low-dose group (*P* < 0.05), while Kir4.1 restoration was more pronounced (*P* < 0.01).

**FIGURE 5:**
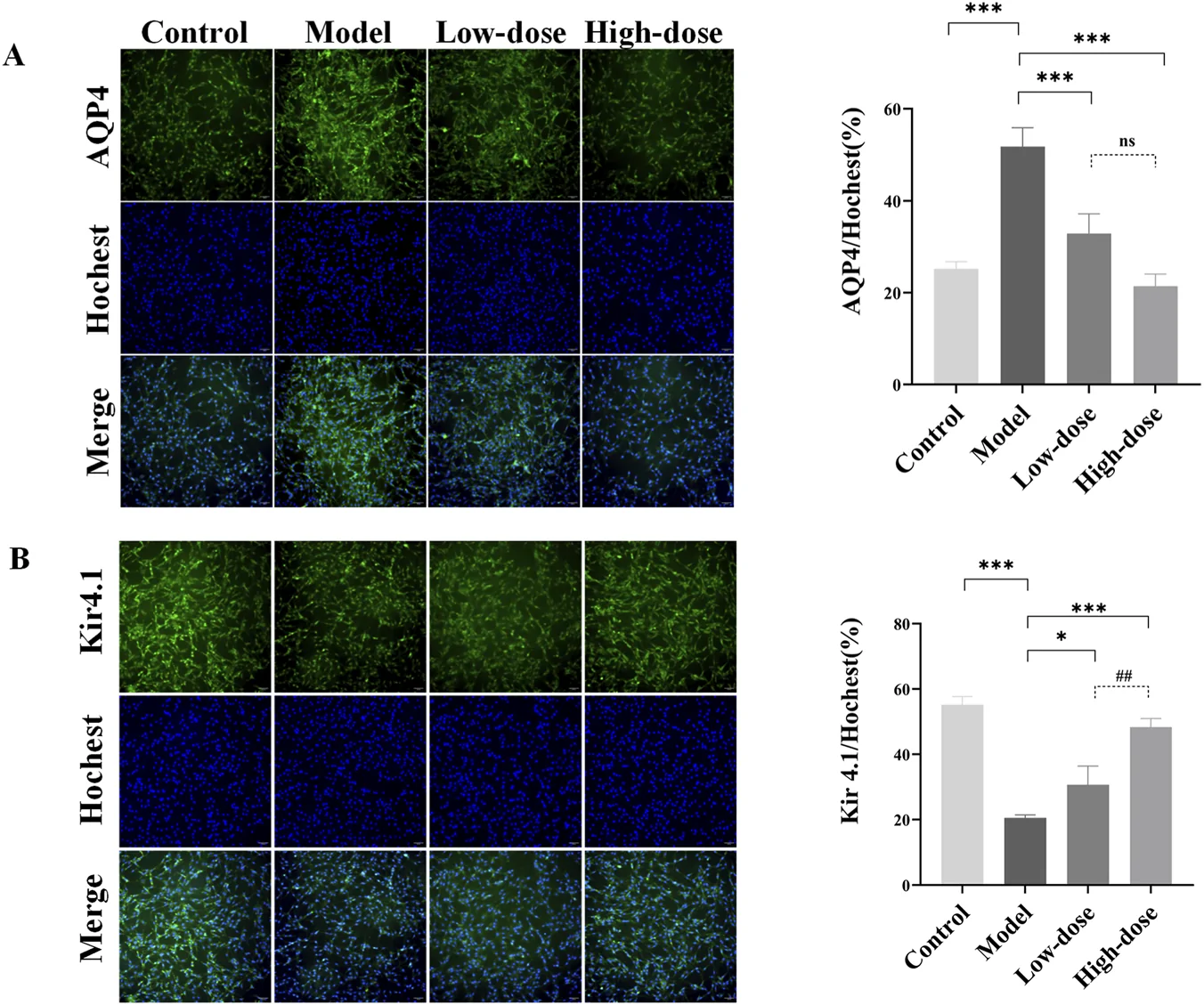
Effects of SCU on AQP4 and Kir4.1 protein expression in HG–induced RMCs. **(A,B)** Representative immunofluorescence images and quantified fluorescence intensity for AQP4 and Kir4.1 across groups. Scale bar: 100 μm. Data are from three independent experiments. Note: vs. control group: *P < 0.05, ***P < 0.001; vs. model group: ^##^ *P* < 0.01.

Western blot analysis (Figure 6A) showed that AQP4 protein levels were significantly elevated in the model group relative to all other groups (all *P* < 0.01), whereas Kir4.1 was markedly suppressed compared to control and both SCU-treated groups (all *P* < 0.001). No significant differences in AQP4 or Kir4.1 protein expression were observed between low- and high-dose groups (all *P* > 0.05).

**FIGURE 6:**
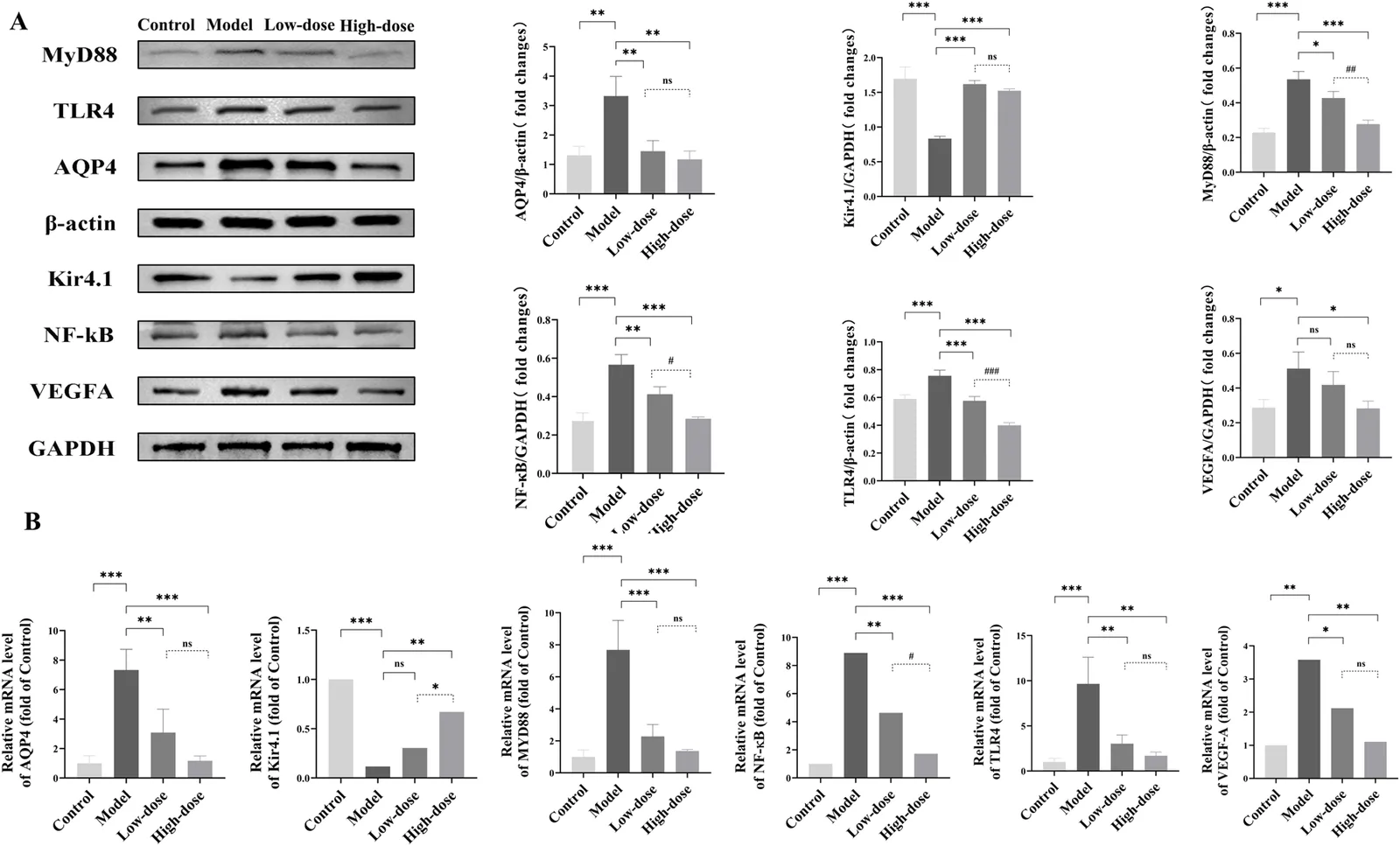
Effects of SCU on inflammation- and edema-associated mediators in HG-stimulated RMCs. **(A)** Western blot analysis and **(B)** qRT-PCR assessment of TLR4, MyD88, NF-κB, VEGFA, AQP4, and Kir4.1 at the protein and transcript levels, respectively. Statistical significance: vs. control group: *P* < 0.05, **P* <0.01, **P < 0.001; vs. low-dose group: ^#^ *P* < 0.05, ^##^ *P* < 0.01, ^###^ *P* < 0.001.

qRT-PCR results (Figure 6B) indicated that AQP4 mRNA was significantly upregulated in the model group versus control and high-dose groups (all *P* < 0.001) and also higher than in the low-dose group (*P* < 0.01). Kir4.1 mRNA was significantly downregulated compared to control (*P* < 0.001) and high-dose (*P* < 0.01) groups. AQP4 mRNA levels did not differ significantly between SCU dose groups (*P* > 0.05), whereas Kir4.1 mRNA was higher in the high-dose than low-dose group (*P* < 0.05).

### 3.6 SCU inhibits the expression of inflammatory cytokines in RMC induced by HG

Western blot and qRT-PCR analyses (Figure 6) revealed that HG markedly upregulated both TLR4 protein and mRNA expression in the model group compared to control and SCU-treated groups (*P* < 0.01–0.001). Notably, SCU exerted a dose-dependent inhibitory effect on TLR4 protein expression. The protein level in the high-dose group was significantly lower than that in the low-dose group (*P* < 0.001), whereas no significant difference was detected at the mRNA transcription level (*P* > 0.05).

MyD88 expression was markedly elevated at both protein and mRNA levels in the model group relative to control and SCU-treated groups (*P* < 0.05–0.001). Moreover, compared with the low-dose SCU group, the high-dose SCU induced a significantly greater reduction in MyD88 protein level (*P* < 0.01), whereas no significant difference was observed at the mRNA transcription level (*P* > 0.05).

NF-κB expression was markedly upregulated at both protein and transcript levels in the model group compared to control and SCU-treated groups (*P* < 0.05–0.001). Notably, high-dose SCU conferred enhanced suppression of NF-κB activation, with both protein and mRNA levels significantly lower than those in the low-dose group (*P* < 0.05), underscoring a dose-dependent inhibitory profile.

Relative to the model group, VEGFA protein expression was significantly reduced in both the control and high-dose groups (*P* < 0.05), while its transcript levels were markedly downregulated across all intervention groups—control, low-dose, and high-dose SCU (*P* < 0.05–0.01).

## 4 Discussion

The pathogenesis of DR is closely linked to inflammation and oxidative stress. Extensive research has substantiated the broad pharmacological properties of SCU, particularly its potent antioxidant capabilities (Wang and Ma, 2018; Nie et al., 2024). In in vitro HG models simulating DR, SCU has been shown to protect the blood-retinal barrier by activating the Nrf2 pathway to scavenge reactive oxygen species (ROS) (Mei et al., 2019). Furthermore, it can directly reduce ROS accumulation induced by HG or hypoxia and block pathological retinal neovascularization by inhibiting the ROS/HIF-1α/VEGF axis (Wang et al., 2014).

The present study confirms that SCU exerts multi-targeted protective effects against HG-induced RMC injury. Under HG conditions, RMCs exhibited significantly decreased cell viability, markedly increased apoptosis, reduced Na^+^-K^+^-ATPase activity, and dysregulated expression of water channels (upregulated AQP4 and downregulated Kir4.1). Concurrently, the TLR4/MyD88/NF-κB/VEGFA signaling pathway was significantly activated, accompanied by a pronounced enhancement in overall cell motility. Notably, SCU intervention dose-dependently reversed these pathological alterations, suggesting its potential for early therapeutic intervention in DR (Figure 7).

**FIGURE 7:**
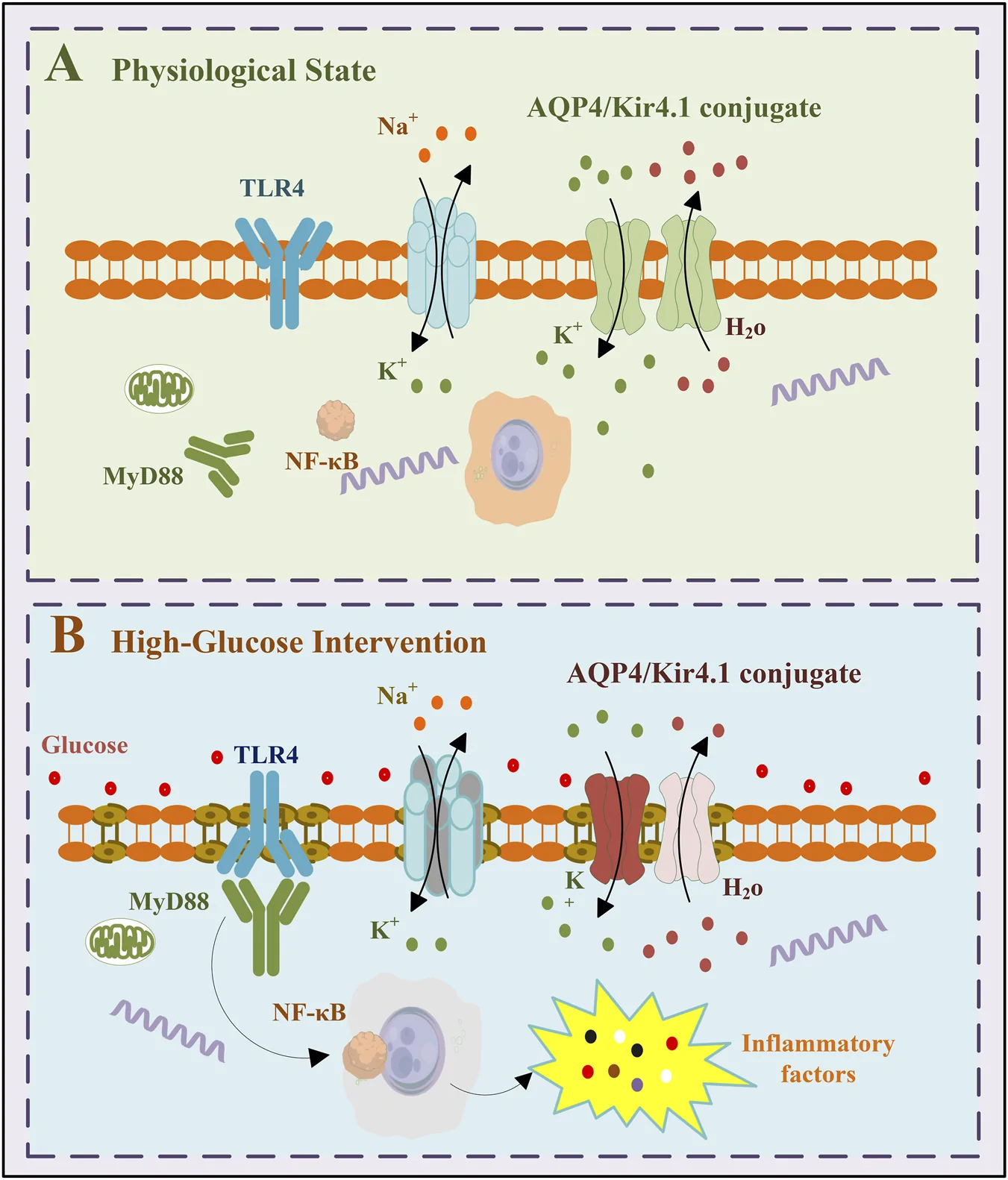
Schematic diagram illustrating the mechanism of HG-induced injury in RMCs and the protective role of SCU. **(A)** Under normal physiological conditions, the AQP4/Kir4.1 complex maintains homeostasis in the retinal microenvironment. **(B)** HG stimulation triggers the uncoupling of AQP4 and Kir4.1, leading to a decrease in Na^+^-K^+^-ATPase activity and the activation of the TLR4/MyD88/NF-κB inflammatory signaling pathway, which ultimately promotes the release of inflammatory cytokines.

Under physiological conditions, RMCs constitute the most abundant glial cell type in the retina (Pereiro et al., 2022; Yang et al., 2022), span the entire thickness of the neural retina with their main processes. These processes connect the inner limiting membrane (ILM, on the vitreal side) and the outer limiting membrane (OLM, on the photoreceptor side). The RMC somata are primarily located in the inner nuclear layer, with the two main processes extending toward the inner and outer aspects of the retina, respectively. The basal end, which extends toward the vitreous, terminates in an expanded, funnel-shaped endfoot. These endfeet line the vitreal surface of the ganglion cell layer and, together with the basement membrane on the inner retinal surface, constitute the ILM. Within the inner and outer plexiform layers, the main processes emit lateral branches that form perisynaptic sheaths enveloping neuronal synapses. The apical main process extends radially outward and gives off numerous microvilli at the level of the OLM, projecting into the subretinal space to ensheath photoreceptors inner segments. The OLM itself is formed by adherens junction complexes between apical Müller cell processes and photoreceptor inner segments. The radial processes of Müller cells provide critical physical scaffolding for the retina, and maintain homeostasis of the retinal metabolic microenvironment by ensheathing neurons, synapses and blood vessels as well as mediating metabolite transport (Reichenbach and Bringmann, 2020).

RMCs participate in retinal glucose metabolism via glycolysis and can absorb and clear neurotransmitters produced by neurons, including glutamic acid, γ-aminobutyric acid, and glycine (Bringmann et al., 2006; Lai et al., 2023). RMCs are rich in various functional proteins, including ion channels, ligand-gated receptors, transmembrane transporters, and membrane-bound enzymes (Beverley and Pattnaik, 2022), which regulate the balance of ions and water across the cell membrane. Light-induced neuronal activity can cause fluctuations in extracellular K^+^ concentration, which are buffered primarily through three mechanisms: the Na^+^-K^+^-ATPase, the synergistic transport of K^+^ with other ions (such as Cl^−^), and K^+^ influx mediated by inward-rectifying potassium channels (Kir) (Lai et al., 2023). Among these, the AQP4 and Kir4.1 proteins form a functional complex and colocalize at the basal endfeet of RMCs at the ILM (Rao et al., 2019; Lai et al., 2023), and form structural associations with the cytoskeletal network composed of β-1-syntrophin and the dystrophin-glycoprotein complex (Rao et al., 2019; Beverley and Pattnaik, 2022), synergistically regulating the osmotic gradient-driven transmembrane water-potassium ion transport process (Gao et al., 2018). Notably, Pannicke et al. (2010) have demonstrated that AQP4 is a critical molecule for maintaining osmotic buffering and cell volume homeostasis in RMC, and its regulatory role in retinal edema is highly context-dependent. At the ILM, AQP4 and Kir4.1 form a functional complex at the RMC endfeet, where they colocalize (Rao et al., 2019; Lai et al., 2023). This complex is structurally linked to a cytoskeletal network composed of β-1-syntrophin and the dystrophin-glycoprotein complex (Rao et al., 2019; Beverley and Pattnaik, 2022), which maintains the polarized expression of AQP4 and Kir4.1 in RMCs (Hirrlinger et al., 2011), and synergistically regulating transmembrane water and K^+^ transport driven by osmotic gradients (Gao et al., 2018). Furthermore, Na^+^-K^+^-ATPase, an integral plasma membrane protein ubiquitously expressed in all eukaryotic cells, hydrolyzes one ATP molecule to actively extrude three Na^+^ ions and import two K^+^ ions against their concentration gradients. The resulting transmembrane ion gradient provides the driving force for various secondary ion transporters. Collectively, the AQP4/Kir4.1 protein complex and Na^+^-K^+^-ATPase provide a crucial foundation for the maintenance of RMC homeostasis.

AQP4 and Kir4.1 in RMCs are closely coupled both structurally and functionally (Goodyear et al., 2010). Under the pathological condition of chronic hyperglycemia in diabetes, this AQP4/Kir4.1 coupling complex is disrupted. AQP4 is significantly upregulated (Chen et al., 2024; Colón-Vázquez et al., 2025), accompanied by a loss of its polarized distribution and a decrease in Kir4.1 expression. This disruption leads to increased water influx and diminished K^+^ buffering capacity, thereby exacerbating cellular edema and inflammation (Fang et al., 2023). HG exposure induces aberrant expression of AQP4 and Kir4.1 and impairs their coupling function, subsequently resulting in cellular edema and BRB damage (Lai et al., 2023). These pathological alterations are consistent with clinical reports showing significantly elevated AQP concentrations and reduced Kir4.1 expression in the aqueous humor of patients with DR (Zhao et al., 2022). In our study, HG exposure similarly led to the upregulation of AQP4, downregulation of Kir4.1, suppression of Na^+^-K^+^-ATPase activity, and increased apoptosis in RMCs, which is highly consistent with previous findings (Fang et al., 2023; Chen et al., 2024; Colón-Vázquez et al., 2025). Importantly, SCU intervention bidirectionally regulates this imbalance. Rather than merely reducing the pathologically elevated AQP4 expression to near-control levels, SCU simultaneously restores the suppressed expression of Kir4.1. This dual action repairs the functional coupling of AQP4 and Kir4.1 and rebuilds the normal osmotic buffering capacity of the cells. These findings suggest that SCU may protect RMCs by maintaining their fundamental homeostasis, highlighting its potential to exert glial protective effects during the early stages of DR.

Under physiological conditions, resting RMCs in the healthy retina are static glial cells lacking migratory capacity. In ischemic retinopathies, RMCs respond to injury through proliferation, migration, and differentiation into a neuron-like phenotype. However, these immediate protective responses can lead to glial scar formation, exacerbating retinal structural damage and disease progression, ultimately resulting in visual impairment (Cutrera et al., 2026; Liu et al., 2026). Previous studies have demonstrated that hyperglycemia, as a core metabolic abnormality in the early stages of DR, can activate RMC migration during gliosis (Li et al., 2020; Tu et al., 2020). Chronic HG exposure may enhance RMC motility by downregulating the gap junction protein Connexin43, thereby impairing intercellular communication, and by inhibiting the transmission efficiency of the Akt signaling pathway (Danesh-Meyer et al., 2016). For instance, Dong et al. (2021) confirmed that a single HG stimulation enhances RMC motility in human primary RMCs, as cross-validated by scratch wound healing and Transwell migration assays. Similarly, Zhou et al. (2023) found that stimulating primary rat RMCs with HG combined with hypoxia for 72 h significantly accelerates scratch wound healing, accompanied by the upregulation of the gliosis marker GFAP. Notably, this phenomenon indicates that cell migration and proliferation do not contradict apoptosis. HG-induced injury in RMCs leads to apoptosis only in a subset of cells, while surviving cells can still initiate a reactive gliosis program, characterized by enhanced motility and compensatory proliferative potential. We also observed enhanced scratch wound healing capacity in RMCs under HG conditions in our study. Importantly, SCU intervention significantly inhibited this HG-induced enhancement in scratch wound healing, suggesting that SCU may interfere with the behavioral alterations associated with RMC glial activation. These findings align well with previous studies demonstrating that SCU can inhibit the proliferation and migration of retinal endothelial cells under HG and hypoxic microenvironments (Long et al., 2019) and alleviate BRB dysfunction and oxidative stress damage caused by microglial activation (Mei et al., 2019). Collectively, these consistencies suggest that SCU may exert inhibitory effects on the abnormal motility and behavioral changes of multiple retinal cell types in a HG environment.

TLR4 is a classic cell surface pattern recognition receptor that activates NF-κB via the MyD88-dependent signaling pathway to mediate inflammatory responses. It can recognize both pathogen-associated molecular patterns and endogenous damage-associated molecular patterns (DAMPs) released during tissue injury, thereby initiating innate immune responses (Akira and Takeda, 2004; Kawasaki and Kawai, 2014). TLRs are stably expressed on the plasma membrane of RMCs (Kumar and Shamsuddin, 2012). Pathological stimuli, such as HG and hypoxia, can aberrantly activate the TLR4/NF-κB signaling pathway in RMCs, contributing to the inflammatory response in DR (Zhou et al., 2023). Activation of this pathway not only directly drives the transcription of pro-inflammatory cytokines, including TNF-α, IL-1β, and IL-6, but also synergistically activates parallel signaling cascades, such as p38 MAPK and Erk (Kumar and Shamsuddin, 2012). This further amplifies the release of chemokines (e.g., CXCL2/MIP-2), exacerbating the retinal inflammatory cascade and microvascular dysfunction (Kumar and Shamsuddin, 2012; Kawasaki and Kawai, 2014). Previous studies have demonstrated that the TLR4-specific inhibitor TAK-242 significantly suppresses the activation of the retinal TLR4/MyD88/NF-κB pathway and the subsequent expression of downstream pro-inflammatory factors in diabetic rat models (Wang et al., 2015; Hu et al., 2017; Bayan et al., 2022). SCU has been shown to alleviate oxidative stress and inflammation by downregulating the TLR4/NF-κB/TNF-α axis, thereby exerting protective effects in renal and neural tissues (Baluchnejadmojarad et al., 2018; Shahmohammadi et al., 2023). In a type 2 diabetic mouse model, SCU has also been confirmed to improve cardiac function by inhibiting the TLR4/MyD88/NF-κB inflammatory pathway (Huo et al., 2021). Furthermore, SCU can directly inhibit NF-κB nuclear translocation and the TLR4/MyD88 cascade, reducing the production of inflammatory mediators such as TNF-α and IL-1β (Wang et al., 2017; Mei et al., 2019; Huo et al., 2021). In our study, we found that HG significantly upregulated the expression of TLR4, MyD88, NF-κB, and downstream VEGFA in RMCs. SCU treatment effectively inhibited the excessive activation of this inflammatory signaling axis, exerting pharmacological effects similar to those of TLR4-specific inhibitors. Recently, Yang et al. (2024) reported that baicalin alleviates the pathological progression of DR by inhibiting the NLRP3 inflammasome. Mechanistically, the activation of the NF-κB pathway serves as a critical priming signal (Signal 1) required for the transcription of NLRP3 and pro-IL-1β (Ting et al., 2008). Therefore, the inhibition of the TLR4/MyD88/NF-κB pathway by SCU may block the inflammatory cascade at an upstream level, potentially preventing the subsequent assembly and activation of the NLRP3 inflammasome. Concurrently, this pathway can also independently drive the expression of downstream pro-angiogenic and inflammatory factors, such as VEGFA, regardless of NLRP3. Collectively, the regulatory effect of SCU on the TLR4/MyD88/NF-κB/VEGFA signaling axis observed in RMCs in this study not only provides supplementary evidence for the regulation of this pathway in DR glial cells but also expands our understanding of the multi-target anti-DR mechanisms of SCU at a more upstream level. This suggests that this pathway may serve as one of the pharmacological targets for the anti-DR effects of SCU.

Our *in vitro* findings provide a mechanistic basis for the role of SCU in DR. The translational relevance of the concentrations used in this study should be comprehensively evaluated from two perspectives: the rationale for *in vitro* concentration selection and the achievable *in vivo* concentrations. The effective concentrations of SCU in this study (25–50 μmol/L) were determined based on gradient screening via CCK-8 assays and are consistent with the effective concentration ranges of SCU in other *in vitro* retinal studies (5–100 μM) (Hu et al., 2019; Su et al., 2025). It is undeniable that SCU has a low oral bioavailability (approximately 10.6% in rats), which poses a critical challenge for its clinical translation (Wang and Ma, 2018). However, existing research has provided proof-of-concept to address this issue. Wang et al. (2017) demonstrated that a Chit-DC-VB12-Scu nanoparticle carrier significantly improved the pharmacokinetic profile of SCU, increasing the AUC by 2–3-fold, and successfully inhibited retinal neovascularization in a DR rat model. This indirectly proves that effective therapeutic concentrations of SCU can be achieved in the retina with the assistance of advanced delivery systems. Therefore, although there is currently a gap between the *in vitro* concentrations used in our study and the *in vivo* exposure levels following standard oral administration, it is feasible to achieve therapeutically relevant concentrations in the retina through the optimization of advanced drug delivery systems. Further *in vivo* translation will require validation combined with optimized pharmacokinetic strategies.

In this study, 100 mM glucose was used to induce RMC injury. Although this concentration exceeds physiological levels, there is currently no universally standardized concentration for *in vitro* DR models. Fang et al. (2023) utilized 50 mM glucose to establish a HG injury model in primary rat RMCs to simulate the pathological microenvironment of DR. Similarly, Chanoumidou et al. (2026), Wei (2022) employed 100 mM glucose to treat human iPSC-derived glial cells, human retinal endothelial cells, and MIO-M1 Müller cells, respectively, to simulate cellular stress and inflammatory responses under diabetic conditions. The selection of 100 mM glucose in our study was based on CCK-8 assay results, as lower concentrations (25–55 mM) failed to induce significant cellular injury in our specific cell batch. This may be attributed to “metabolic adaptation” in cells maintained in HG culture environments over the long term (Chanoumidou et al., 2026), necessitating higher glucose concentrations to overcome this adaptation and induce an acute stress state within a short timeframe that is comparable to chronic metabolic dysregulation *in vivo*. This model successfully induced the AQP4/Kir4.1 imbalance and the activation of the TLR4/MyD88/NF-κB/VEGFA inflammatory axis, which are highly consistent with clinical DR pathologies, thereby providing a reliable molecular phenotypic platform for evaluating the protective effects of SCU under HG stress.

The use of 100 mM glucose in this study inevitably introduces hyperosmotic stress. However, from the perspective of DR pathophysiology, hyperglycemia and hyperosmolarity are not two independent injurious factors. Hyperglycemia leads to intracellular sorbitol accumulation via the polyol pathway, forming an endogenous hyperosmotic state, which is itself an integral metabolite of the DR pathogenic mechanism (Zapuskalov et al., 2000). Furthermore, Chen et al. (2012) employed gradient concentrations of 25 mM and 100 mM glucose with isosmotic mannitol controls in ARPE-19 cells; they demonstrated that mannitol, under equivalent osmotic pressure, failed to replicate the proteomic and oxidative stress alterations mediated by HG, confirming the existence of specific toxicity of HG independent of osmotic pressure. In our study, Western blot results showed that 100 mM glucose can stably activate the TLR4/MyD88/NF-κB/VEGFA inflammatory pathway, an effect that is difficult to explain solely by hyperosmotic stress. Therefore, the pathological changes observed in this study are likely the result of a cascade of reactions driven by the combined effects of glucose toxicity and hyperosmotic stress, which is consistent with the perspective proposed by Mühle et al. (2025) that HG and hyperosmolarity synergistically amplify tissue injury. Thus, 100 mM glucose in this study constitutes a mixed stress condition of “HG combined with hyperosmolarity”.

Although previous studies have demonstrated that SCU can exert protective effects on the retinal barrier by inhibiting the NLRP3 inflammasome (Yang et al., 2024), regulating the VEGF/Ang/Tie2 pathway to suppress pathological neovascularization (Ding et al., 2025), and activating the Nrf2/HO-1/NQO1 pathway to scavenge ROS (Mei et al., 2019), these investigations have primarily focused on *in vivo* models and retinal vascular endothelial cells. The present study provides the first evidence that, under HG conditions, SCU restores the expression balance of AQP4/Kir4.1 and Na^+^-K^+^-ATPase activity in RMCs. This suggests that SCU may regulate the expression balance of proteins associated with water and ion homeostasis, thereby contributing to the maintenance of osmotic homeostasis in the retina. Furthermore, SCU dose-dependently modulates the expression of the TLR4/MyD88/NF-κB/VEGFA inflammatory signaling pathway in RMCs under HG conditions, while also inhibiting their aberrant cell motility and apoptosis. Collectively, SCU exerts multi-faceted protective mechanisms in RMCs, which are crucial for retinal homeostasis, highlighting its potential for early therapeutic intervention in DR.

However, while this study focuses on elucidating the intrinsic mechanisms of action of SCU, a structurally well-defined flavonoid metabolite, in physiologically relevant primary cell models, it still has some limitations. First, the downstream mechanistic experiments lacked osmotic controls, making it impossible to strictly distinguish glucose-specific effects from hyperosmotic effects at the molecular level. Future studies should incorporate isosmotic mannitol controls in downstream assays to further elucidate the independent contributions of osmotic pressure and metabolic toxicity. Second, the absence of pathway blockade strategies, such as TLR4 inhibition or siRNA knockdown, means that the causal relationship between SCU and the TLR4/MyD88/NF-κB pathway has not been directly validated. Subsequent research should employ TLR4-specific inhibitors (e.g., TAK-242), siRNA knockdown, or rescue experiments to definitively establish the causal regulatory role of SCU in this pathway. Third, while the enhanced scratch wound healing capacity of RMCs under HG conditions may involve both cell migration and proliferation, this study did not use mitotic inhibitors, such as mitomycin C, to differentiate between the two. Therefore, the respective effects of migration and proliferation could not be strictly isolated. Fourth, all experiments were conducted using an *in vitro* primary RMC culture model. The pharmacodynamic and pharmacokinetic profiles of SCU *in vivo* in diabetic animal models remain to be verified. Furthermore, future research must include standard positive drug controls (e.g., TAK-242, aflibercept) to rigorously evaluate the direct comparison between SCU and existing therapies, thereby providing more reliable dosing references for clinical translation. Fifth, future studies should validate the direct effects of SCU on oxidative stress markers (e.g., ROS, Nrf2) in RMCs under HG conditions and assess downstream inflammatory cytokines, such as TNF-α, IL-1β, IL-6, or MCP-1, to provide stronger evidence for its anti-inflammatory activity. Finally, the direct anti-edema function of SCU should be further validated through experiments measuring cell volume, water permeability, or *in vitro* barrier function. A systematic evaluation of the therapeutic potential of SCU for DR will require the continued in-depth validation of these mechanisms.

## 5 Conclusion

The early stages of DR can be considered NUV rather than merely microvascular disease. From the perspective of glial cell protection within the NVU, this study demonstrates that SCU exerts significant multi-targeted protective effects against HG-induced RMC injury. On the one hand, SCU effectively reverses RMC apoptosis and aberrant hypermotility, and restores the expression balance of AQP4/Kir4.1 proteins and Na^+^-K^+^-ATPase activity, suggesting its role in maintaining retinal osmotic homeostasis and water-ion balance. On the other hand, SCU dose-dependently downregulates the excessive activation of the TLR4/MyD88/NF-κB/VEGFA inflammatory signaling pathway, potentially mitigating HG-induced inflammatory damage. The concurrent improvement in these phenotypic and molecular markers indicates that SCU possesses multi-targeted pharmacological properties, including the maintenance of water and ion homeostasis, the alleviation of inflammatory injury, and glial cell protection. These findings provide an experimental basis for SCU as a candidate therapeutic agent for delaying DR progression. Future studies will further validate these mechanisms and evaluate the clinical translational potential of SCU by combining *in vivo* DR models with specific pathway interventions.

## Data Availability

The datasets presented in this study can be found in online repositories. The names of the repository/repositories and accession number(s) can be found in the article/Supplementary Material.
