# Supplementary material for: Scutellarin attenuates high glucose-induced edema and inflammation in retinal Müller cells via AQP4/Kir4.1 restoration and TLR4/MyD88/NF-κB inhibition
Source: Front Pharmacol. 2026 Sep 18;17:1923023. doi: 10.3389/fphar.2026.1923023 (PMC13630717; doi:10.3389/fphar.2026.1923023)
Supplement: Supplementary file 1 [file DataSheet1.pdf]

产品分析证书

Certificate of Analysis

产品信息（Information）

中文名称(ChineseName)	野黄芩苷	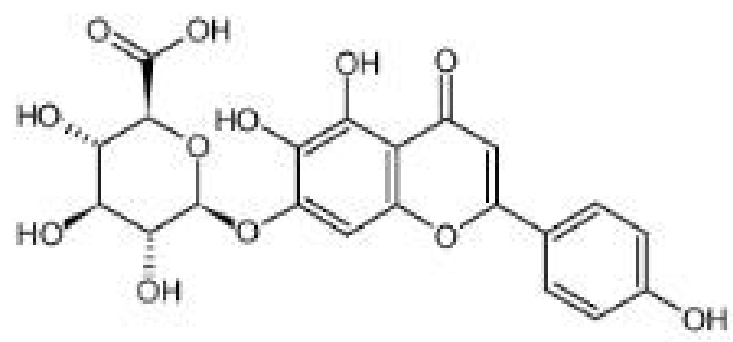
英文名称(Product Name)	scutellarin	
产品编号(Product No.)	AB1174	
CAS Number	27740-01-8	
分子式(M. F.)	C21H18O12	
分子量(M. W.)	462.36	
批　号(Lot No.)	AFBL0861	

分析结果（Analytical result）

检测项目(Test Item)	质量标准(Specifications)	结果(Results)
外　观(Appearance)	Dark yellow powder	Conforms
纯　度(Purity)	≥98.0%	98.84%
质　谱(MS)	Comply with the structure	Conforms
核　磁(NMR)	Comply with the structure	Conforms
干燥失重(Loss on drying)	＜3.0%	Conforms
结　论(Conclusion)	Conforms to enterprise standards	

注意事项（Warm Notice）

储　存(Storage)	Sealed storage in a cool and dry place(2-8°C)，avoid light
使用方法(Use)	Freshly preparing the solution before using.For lab research use only and can't be used for human
备　注(Reminder)	If any quality problem，please contact us within 15 days after receiving
温馨提示(Note)	When publishing,please cite as： **Chemical Name** was purchased from **Chengdu Alfa Biotechnology Co.Ltd.(Chengdu,China)**

Q C：He Bin

Date: ______

HPLC

Instrument: Waters-HPLC **Column:** C18，4.6*250 mm，5 μ m

Detection Mode: 335nm **Column temperature:** 35℃

Flow Rate: 1.0ml/min **Sample dissolution:** Methanol

Mobile Phase: Methanol ：0.1%Phosphoric acid in water=40：60

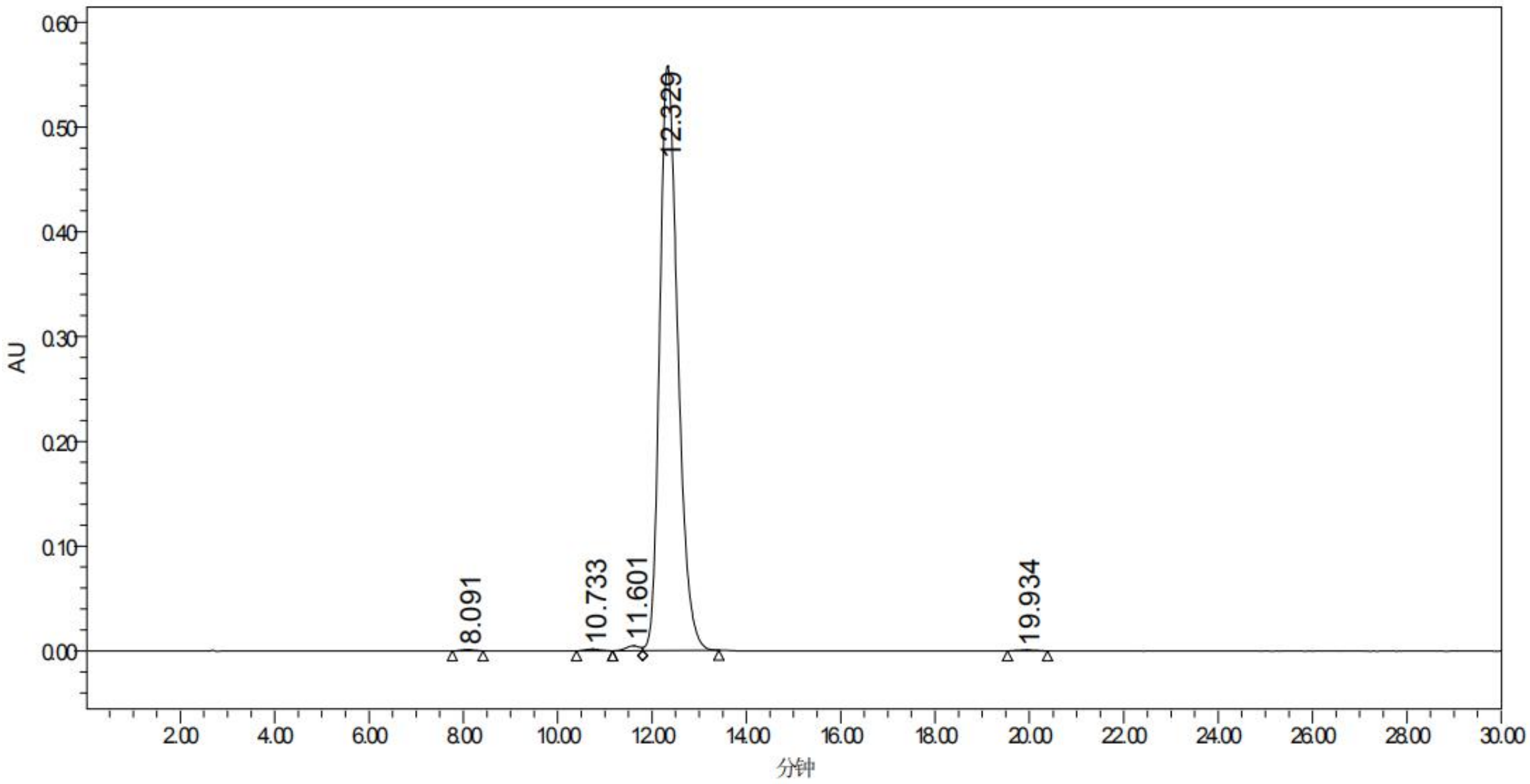

	名称	保留时间	面积	%面积
1		8.091	23996	0.16
2		10.733	33969	0.23
3		11.601	93762	0.62
4		12.329	14866942	98.84
5		19.934	22442	0.15
