# Supplementary material for: Scutellarin attenuates high glucose-induced edema and inflammation in retinal Müller cells via AQP4/Kir4.1 restoration and TLR4/MyD88/NF-κB inhibition
Source: Front Pharmacol. 2026 Sep 18;17:1923023. doi: 10.3389/fphar.2026.1923023 (PMC13630717; doi:10.3389/fphar.2026.1923023)
Supplement: Supplementary file 2 [file Table1.docx]

Supplementary Material

# Supplementary Tables

**Table 1 Dilution ratios for primary antibodies in immunofluorescence staining.**

| Protein | Species | Dilution | Company | Catalog No |
| --- | --- | --- | --- | --- |
| AQP4 | Rabbit | 1:100 | Proteintech Group，Inc | 16473-1-AP |
| Kir4.1 | Rabbit | 1:200 | Proteintech Group，Inc | 12503-1-AP |
| Vimentin | Rabbit | 1:200 | Abcam | ab92547 |
| GS | Rabbit | 1:200 | Proteintech Group，Inc | PTG-11037-2-AP-50ul |
| CRALBP | Rabbit | 1:50 | HuaBio | HA721330 |

**Abbreviations**: GS: Glutamine Synthetase ; CRALBP: Cellular retinaldehyde-binding protein.

**Table 2 Dilution ratios for primary antibodies in western blotting.**

| Protein | Species | Dilution | Company | Catalog No |
| --- | --- | --- | --- | --- |
| AQP4 | rabbit | 1:5000 | Proteintech Group，Inc | 16473-1-AP |
| Kir4.1 | rabbit | 1:5000 | Proteintech Group，Inc | 12503-1-AP |
| VEGFA | rabbit | 1:5000 | Proteintech Group，Inc | 19003-1-AP |
| TLR4 | rabbit | 1:5000 | Proteintech Group，Inc | 19811-1-AP |
| MyD88 | rabbit | 1:5000 | Proteintech Group，Inc | 23230-1-AP |
| NF-κB | rabbit | 1:5000 | Proteintech Group，Inc | 10745-1-AP |

**Table 3 List of PCR primer sequences.**

| Gene | Forward | Reverse |
| --- | --- | --- |
| GAPDH | GCATCTTCTTGTGCAGTGCCCCGACC | TACGGCCAAATCCGTTCACATCAC |
| VEGF-A | CGGGCCTCTGAAACCATGAA | GCTTTCTGCTCCCCTTCTGT |
| TLR4 | TCCAGAGCCGTTGGTGTATC | GAAGATGTGCCTCCCCAGAG |
| NF-KB | AAGACAGCACATAGATGAGCTCCG | ATAGCAGTGGGCCATCTCCA |
| MyD88 | GTTCTCTCTACCGTTGGTCGC | CATCCAACAAACTGCGAGTGG |
| AQP4 | AAACCCCAGAAGACAGCACC | GGGAGGTCCACACTTACAGC |

| Kir4.1 | TTCATCTGTCCCACTACCCC | CTGCGTCGTCTGGCTGTAATA |
| --- | --- | --- |
